# Nephrocutaneous Fistula due to Xanthogranulomatous Pyelonephritis

**DOI:** 10.7759/cureus.3467

**Published:** 2018-10-19

**Authors:** Simcha Weissman, Madeeha Ghaffar, Dana Safavian, Sharma Rubal, Anthony Khabut, Mohammad G Maruf, Michael Krzyzak

**Affiliations:** 1 Internal Medicine, Touro College of Osteopathic Medicine, New York, USA; 2 Internal Medicine, Staten Island University Hospital, Staten Island, USA

**Keywords:** fistulae, xanthogranulomatous pyelonephritis (xgp), atrophic kidney, proteus mirabilis

## Abstract

While the development of a fistulous tract from the kidney to the proximal adjacent organs is relatively common, a tract leading to the skin is a rare occurrence. The primary cause of a fistula is prior surgical intervention or malignancy leading to abscess formation. Our case involves Xanthogranulomatous pyelonephritis (XGP) causing a longstanding lobulated abscess, ultimately leading to the formation of a fistulous tract.

## Introduction

Purulent materials tend to drain toward the path with least resistance, which is generally the bowel or some other local organ. Our case is rare since the fistula developed a path toward the skin, which in our bed-bound patient was indeed very low in resistance. Additionally, a major underlying cause of the renal fistulae development is previous surgery to an adjacent body region, such as the hip, as well as a nephrectomy [1]. In our case, the tract was due to Xanthogranulomatous pyelonephritis (XGP) causing a longstanding lobulated abscess. These events were presumed to be caused by a *Proteus mirabilis* infection allowing bacteria to multiply, ultimately leading to severe inflammation and kidney atrophy, making our case a true rarity. This presentation is extremely dangerous as longstanding abscess and infection can lead to atrophy of the involved organ and eventual sepsis [2].

## Case presentation

**Figure 1:**
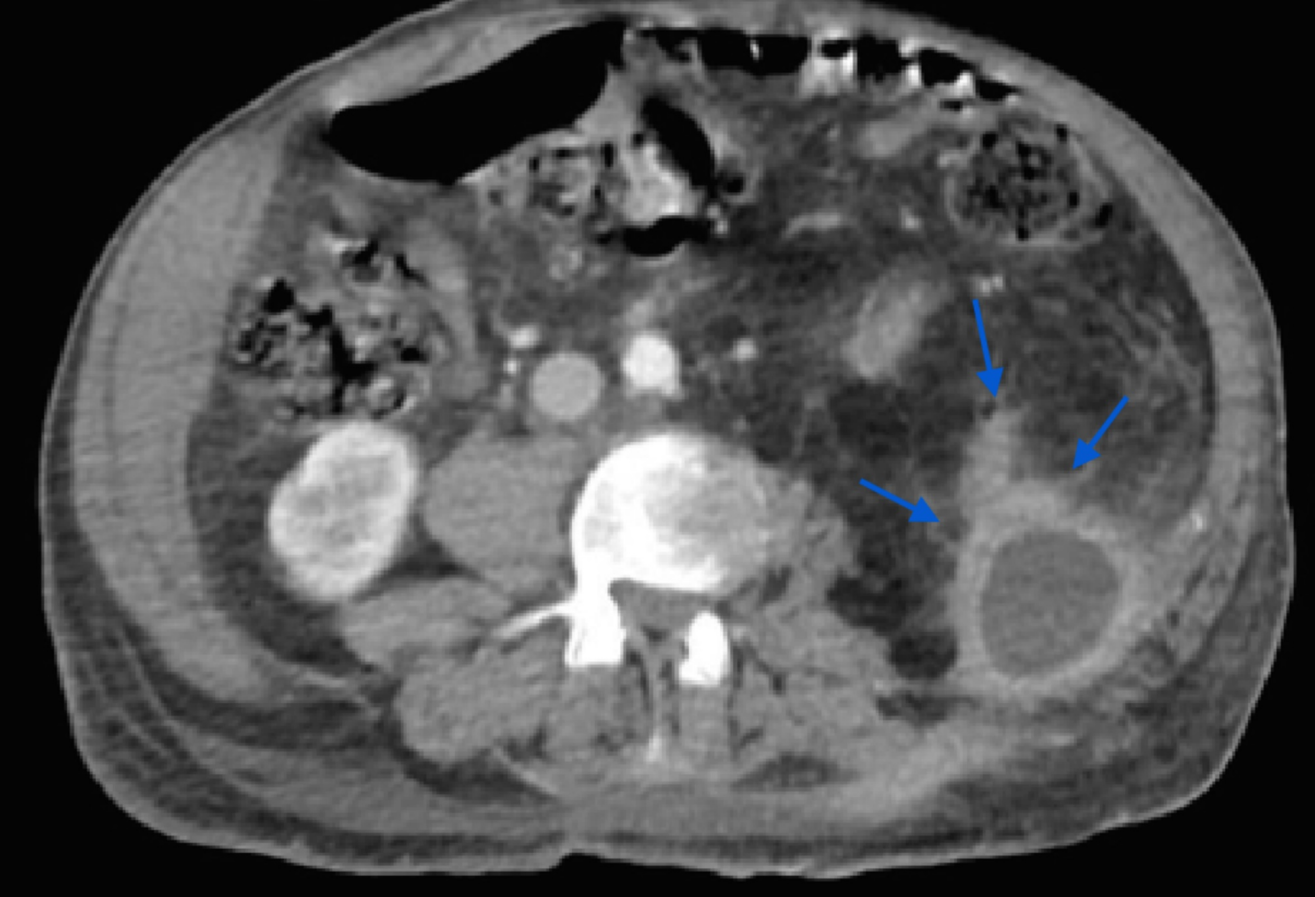
CT scan of the abdomen and pelvis illustrating fluid collection along with severe inflammation of the left kidney CT: computed tomography

**Figure 2:**
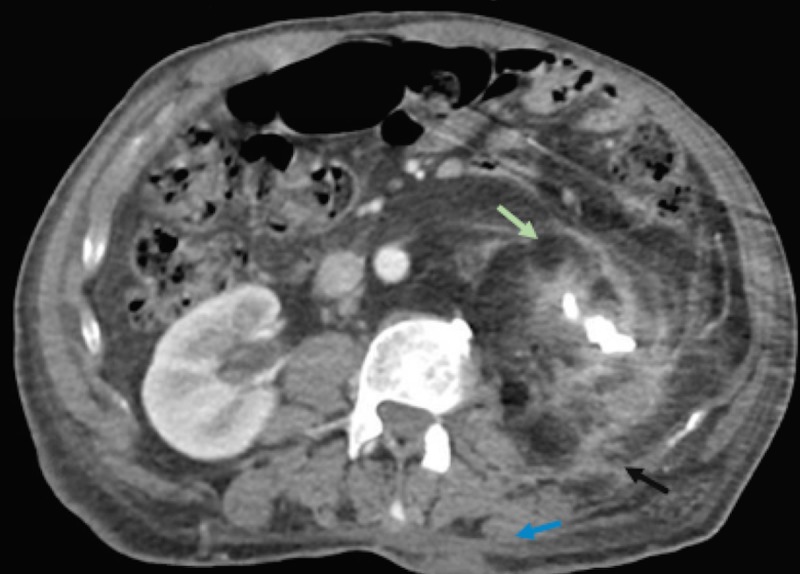
CT scan of the abdomen and pelvis displaying an atrophic left kidney (green arrow), fistula to the adjacent tissue (black arrow), as well as a fistula to the skin (blue arrow) CT: computed tomography

**Figure 3:**
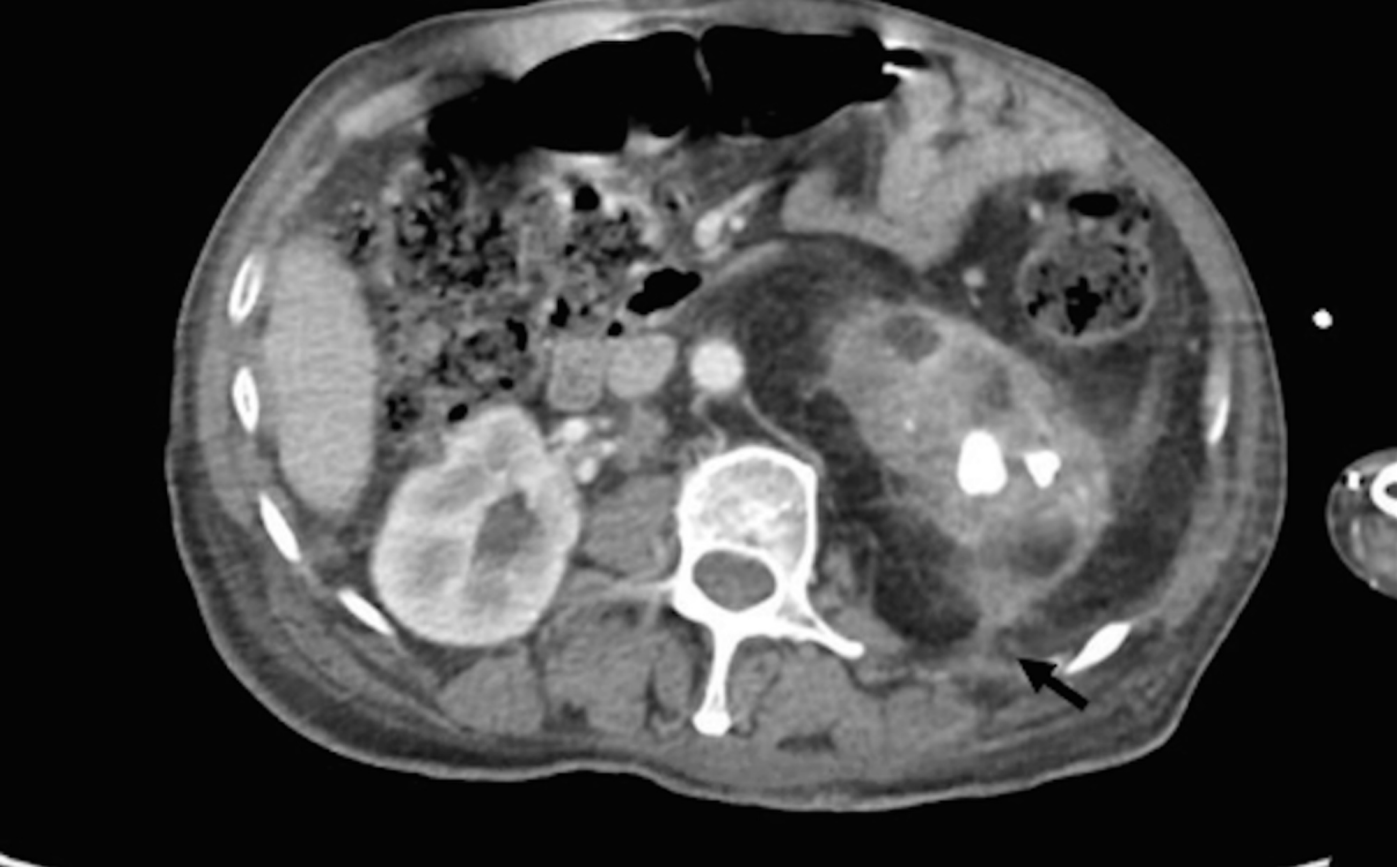
CT scan showing a fistulous tract involving the para-spinal muscles CT: computed tomography

**Figure 4:**
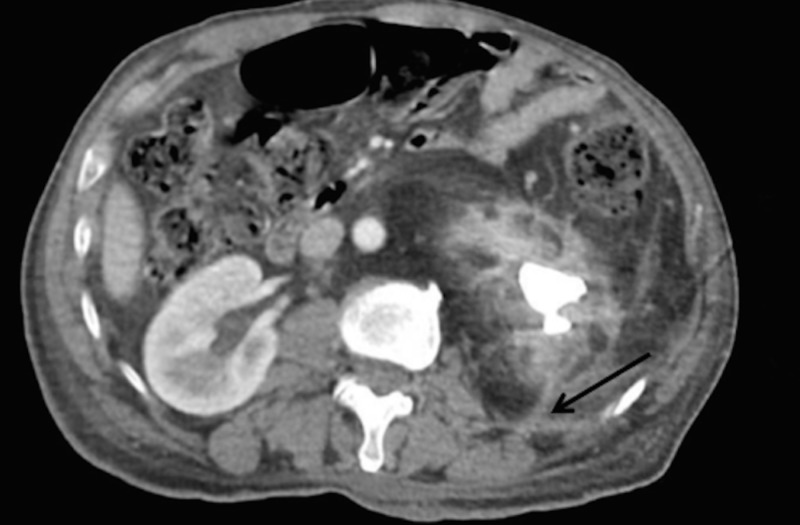
CT scan clearly delineating the formation of a nephrocutaneous fistulous tract

Our patient is a bed-bound 57-year-old male with a past medical history of Down's syndrome and quadriplegia. He was sent to the emergency department (ED) by his primary care provider (PCP) for being hypotensive and febrile. The patient had multiple episodes of urinary tract infections (UTIs) over the course of several years, but this time he was found to be septic. On admission, he was afebrile with a blood pressure of 97/64 and a heart rate of 89 bpm. On the medicine floors, urine analysis (UA) confirmed moderate blood in the urine along with a positive leukocyte esterase, confirming a urinary tract source. Urine and blood cultures were drawn and turned out positive for *Proteus mirabilis* in the urine. This complicated his hospital stay as XGP was eventually diagnosed as the outcome of his longstanding bacteriuria. During a routine physical exam by the medicine night team, what appeared to be a pressure ulcer was identified on our patient’s lumbar spine. Non-contrast computed tomography (CT) showed a highly atrophic left kidney with visible inflammation surrounded by a collection of fluid (Figure 1). Additionally, a peri-nephric abscess and a 3.5-cm staghorn calculi were readily seen. A contrast CT showed a fistulous tract from the left kidney through the para-spinal muscles of the back and into the lumbar spine (Figures 2-4). Infectious disease and urology consultations recommended intravenous (IV) ampicillin and ciprofloxacin. The patient underwent incision and drainage by interventional radiology and had a Jackson-Pratt (JP) drain inserted. IV fluids were given to return the blood pressure to the baseline. Additionally, a total course of six weeks of antibiotic therapy was recommended by urology to cool down the inflammation until a left nephrectomy would eventually be performed.

## Discussion

XGP is an unusual form of chronic pyelonephritis associated with indolent bacterial infection. The disease begins in the renal pelvis and then proceeds to extend into the medulla and cortex, eventually involving the renal parenchyma. During this disease process, the parenchyma is destroyed and replaced with lipid-laden macrophages. *Proteus mirabilis* infection accounts for less than 1 % of chronic pyelonephritis. It usually affects middle-aged women with long-term diabetes and recurrent kidney stones [3]. Most cases of XGP are unilateral; however, bilateral disease has also been reported and is generally fatal. If uncontrolled, it can spread to the adjacent tissues [4]. Patients usually present with systemic features, including malaise, fever, chills, weight loss, along with urinary complaints of flank pain, increased frequency of micturition, dysuria, and nocturia.

Three forms of XGP are recognized: diffuse – characterized by diffuse involvement of the kidney, segmental – characterized by segmental involvement, and focal – which is located within the cortex. No clinical or radiologic features are diagnostic of XGP. It is often misdiagnosed pre-operatively as pyelonephritis, tuberculosis, peri-nephritic abscess, and renal cell carcinoma (RCC) [4-5]. Complications can occur in the form of psoas abscess, nephrocutaneous fistula, entero-colonic fistula, para-nephritic abscess, and sepsis. Ultrasound of XGP typically demonstrates an enlarged kidney with a large amorphous central echogenicity that corresponds to a renal pelvis staghorn calculus, multiple fluid-filled masses, and pelvic contracture [6]. Abdominal X-ray shows renal calculi. Contrast-enhanced CT is a reliable method to establish the presence and extent of extra-renal involvement, leading to the diagnosis of XGP. The common CT findings of XGP include calculi, hydro-nephrosis, and hypo-dense areas with the focal areas of parenchymal destruction filled with purulent materials such as pus [7]. Magnetic resonance imaging can be a valuable tool due to its ability to identify the accumulation of lipid-laden foamy macrophages. Hence, while our case was identified via contrast-induced CT, there are a number of methods that allow for visualization of the underlying pathologic state. 

## Conclusions

XGP accompanied by complications, such as longstanding renal stones, requires an immediate evaluation and early diagnosis. Our case illustrates prior surgery is not the only cause of fistulae. Diagnosis was difficult to elucidate because of the patient’s mental status due to Down's syndrome and his bedridden ambulatory state; thus, his renal symptomatology was previously thought to be due to recurrent urinary tract infections. The intent of this case is to make medical and surgical staff aware that a cutaneous lesion, albeit looking like a pressure ulcer, may be the first clinical phenomenon of an abscess collection, fistulous tract, and pending atrophic kidney. Furthermore, one should be more cautious of this phenomenon in a bed-bound patent as the path of least resistance is in fact to the skin.
